# One‐pot inimer promoted ROCP synthesis of branched copolyesters using α‐hydroxy‐γ‐butyrolactone as the branching reagent

**DOI:** 10.1002/pola.28048

**Published:** 2016-02-11

**Authors:** Geng Hua, Johan Franzén, Karin Odelius

**Affiliations:** ^1^ Department of Fibre and Polymer TechnologyKTH Royal Institute of TechnologyStockholmSE‐100 44Sweden; ^2^ Department of ChemistryKTH Royal Institute of TechnologyStockholmSE‐100 44Sweden

**Keywords:** γ‐butyrolactone, branched, inimer, kinetics (polym.), polyesters, polymerization kinetics, ring‐opening copolymerization

## Abstract

An array of branched poly(ɛ‐caprolactone)s was successfully synthesized using an one‐pot inimer promoted ring‐opening multibranching copolymerization (ROCP) reaction. The biorenewable, commercially available yet unexploited comonomer and initiator 2‐hydroxy‐γ‐butyrolactone was chosen as the inimer to extend the use of 5‐membered lactones to branched structures and simultaneously avoiding the typical tedious work involved in the inimer preparation. Reactions were carried out both in bulk and in solution using stannous octoate (Sn(Oct)_2_) as the catalyst. Polymerizations with inimer equivalents varying from 0.01 to 0.2 were conducted which resulted in polymers with a degree of branching ranging from 0.049 to 0.124. Detailed ROCP kinetics of different inimer systems were compared to illustrate the branch formation mechanism. The resulting polymer structures were confirmed by ^1^H, ^13^C, and _1_H‐_13_C HSQC NMR and SEC (RI detector and triple detectors). The thermal properties of polymers with different degree of branching were investigated by DSC, confirming the branch formation. Through this work, we have extended the current use of the non‐homopolymerizable γ‐butyrolactone to the branched polymers and thoroughly examined its behaviors in ROCP. © 2016 The Authors. Journal of Polymer Science Part A: Polymer Chemistry Published by Wiley Periodicals, Inc. J. Polym. Sci., Part A: Polym. Chem. **2016**, *54*, 1908–1918

## INTRODUCTION

Introducing branches to polymers provides materials with distinctly different properties compared to analogous linear polymers, including both a reduction in viscosity and an increase in flexibility.^1^ For aliphatic polyesters, the abundance of terminal‐groups inherent to branched polymers also strongly affects the degradation profile of the material,^2^ leading to an altered degradation rate depending on the nature and amount of the end‐groups. These properties have been studied and reviewed in several recent publications.^3^, ^4^, ^5^, ^6^, ^7^

Numerous routes for achieving branched or hyperbranched structures have been applied to synthesize aliphatic polyesters.^8^ Polycondensation is the most conventional route, and branched polymers are commonly synthesized via the polycondensation of AB_*n*_ type of monomers. These condensation studies have been well developed with a broad polymer family that includes polyesters,^9^, ^10^, ^11^ polyamides,^12^, ^13^ polyethers,^14^ and polycarbonates.^15^ Another strategy that was introduced in mid 1980s to early 1990s by several different groups described the use of a latent AB_n_ monomer in a type of multibranching polymerization.^16^, ^17^, ^18^, ^19^, ^20^, ^21^ Such monomers behave both as a monomer and as an initiator and are thus termed inimers. Inimer‐promoted hyperbranched polymers comprise a smaller family of materials than regular AB_*n*_‐type of monomers. Advantages of using inimers include better control over the molecular weight and distributions, an overall control of the branching polymerization and lower risk of gelation.

To extend the current use of bioresourced cyclic monomers and to introduce the very limited studied 5‐membered lactones to branched polymers, we hereby investigated the commercially available α‐hydroxy‐γ‐BL (αOHγBL) as the inimer to create branched polyesters. Ring‐opening polymerization (ROP) studies of 5‐membered cyclic γBL and its derivatives were first reported decades ago for forming linear polymers.^22^ The γBLs are incapable of undergoing ring‐opening homopolymerization to obtain high molecular weight polymers under common lab‐accessible conditions.^23^ Despite the poor ability of ROP for γBLs, ring‐opening copolymerization (ROCP) with other lactones is favorable. In the ROCP strategies, the γBLs are combined with a higher strained lactone such as ɛ‐caprolactone (ɛCL) and a number of studies have successfully conducted the ROCP of β‐propiolactone‐γBL,^24^ tetramethylene urea‐ γBL,^25^ glycolid‐γBL, and ɛCL‐γBL,^26^ α‐methylene‐γBL‐ɛCL,^27^ and α‐bromo‐γBL‐ɛCL.^28^ For most of these copolymerizations, a relatively low conversion of the five‐membered monomer was observed. To obtain copolymers with more γBL units, an initial excess amount of the monomer is required.

There exist elegant examples of inimer‐promoted ROCP of branched poly‐ɛCL. For instance, 4‐(2‐hydroxyethyl)‐ɛ‐caprolactone was synthesized and used as an inimer to form highly branched poly(ɛ‐caprolactone).^29^ Another reported example using bis(hydroxymethyl)‐substituted ɛCL as the inimer for ring‐opening polymerization to form hyperbranched polyesters.^30^ This same inimer was also copolymerized with ɛCL, and the resulting copolymer was used as a nanoporosity template‐agent for organosilicates.^31^ Other examples of degradable branched copolymers such as 5HDON‐poly(glycolide),^32^ polycarbonate,^33^ and polylactide^34^ all show very interesting results with varied material properties. These reported systems exhibited good control over the hyperbranched structures and have broadened the study of hyperbranched copolymers both in synthesis and characterizations. However, the preparation of inimers usually involves multiple‐step reactions. Thus, to move towards the current trend in sustainability, more straightforward synthesis routes utilizing commercially available or bioresourced inimers and monomers need to be developed.

Hence, αOHγBL was chosen as a means towards the goal. Upon successful ROCP of the inimer with ɛCL, the use of five‐membered lactones is extended beyond linear copolymers, which has not yet been achieved. The low ring‐strain of the γBL directly results in a lower reactivity than ɛCL, which favors the dispersed existence of branching points along the polymer chain, instead of branch formation in clusters. Therefore, the aim was to build a statistically branched copolyester system through an inimer‐promoted strategy to broaden the use of γBLs in polyesters under facile reaction conditions, to elucidate the polymerization process of multibranching polyesters, and to study the physical properties of the resulting polyesters.

## EXPERIMENTAL MATERIALS

2‐Hydroxy‐gamma‐butyrolactone (αOHγBL, Acros Organics, 99%), DL‐α‐Hydroxyl‐β,β‐dimethyl‐γ‐butyrolactone (αOHβMe_2_γBL, Fluka, 98%), benzyl alcohol (BnOH, Sigma‐Aldrich, 99.8%), toluene (Acros Organics, extra dry 99.85%) were used as received without further purification. ɛ‐Caprolactone (ɛCL, Aldrich, 97%) was dried over CaH_2_ (Sigma‐Aldrich, 95%) for 48 h and distilled under reduced pressure. Stannous octoate (Sn(Oct)_2_, Aldrich, 92.5–100%) was dried over 3 Å molecular sieves prior to use.

### Polymerization Procedures

#### General Inimer‐ɛCL Ring‐Opening Copolymerization

All glasswares employed in the ROP reactions were oven dried at 150 °C for over 48 h prior to use. For the general ROCP in bulk, the desired amount of ɛCL (1.72 g, 15.1 mmol), αOHγBL (154.1 mg, 1.51 mmol) or αOHβMe_2_γBL (196.5 mg, 1.51 mmol) and Sn(Oct)_2_ (16.8 mg, 0.042 mmol) were weighted into a 25 mL round bottom flask equipped with a PTFE stirring bar in a N_2_ atmosphere glove box (Mbraun MB 150‐GI). The sealed flasks were then placed in a preheated 110 °C silicon oil bath, and *t* = 0 was noted as the moment when the glassware was placed into the oil bath. After 18 to 60 h, the flask was taken out of the oil bath and was rapidly cooled to room temperature. The resulting product was dissolved in chloroform and precipitated into an excess amount of cold methanol twice and diethyl ether once. The precipitates were then dried under reduced pressure until stable weights, sealed and kept in 4 °C fridge until further characterizations.

#### Kinetic Study of Inimer‐ɛCL ROCP

An oven‐dried 2‐neck 25 mL round bottom flasks was charged with desired amount of inimer, that is, αOHγBL (154.1 mg, 1.51 mmol) or αOHβMe_2_γBL (196.5 mg, 1.51 mmol), ɛCL (1.72 g, 15.1 mmol), and Sn(Oct)_2_ (6.7 mg, 0.017 mmol) in an N_2_ atmosphere glove box. Benzyl alcohol (18.0 mg, 0.17 mmol) was added to a control group as a co‐initiator. The reactants were diluted to [CL + inimer]_0_ = 1 M in extra dry toluene. The reaction was started by lowering it into a preheated 110 °C silicon oil bath using *t* = 0 as the moment when the glassware was placed in the oil bath. Aliquots were withdrawn using a syringe with a needle under an N_2_ atmosphere at defined time intervals. The solvent was removed via evaporation, and the residues were sealed and maintained in a refrigerator at 4 °C until further characterizations. After 24 h to 72 h, the flask was taken out of the oil bath and was cooled to room temperature. Toluene was removed under reduced pressure, and the resulting products were dissolved in chloroform then precipitated twice in excess cold methanol.

### Instrument

#### Size Exclusion Chromatography (SEC)

Number average molar mass (*M*_n_) and dispersity (Ð) of the resulting copolymers were determined using a Verotech PL‐GPC 50 Plus system equipped with a PL‐RI detector and two Mixed‐D (300 × 7.5 mm) columns (Varian, Santa Clara). The samples were injected using a PL‐AS RT Autosampler. Chloroform was used as the mobile phase at an injection rate of 1 mL/min at 30 °C, and toluene was used as the internal standard for flow rate fluctuation corrections. Polystyrene standards with a narrow mass distribution and a molecular weight of 160–371,000 g/mol were used for calibration.

#### Triple Detector SEC

Triple detector SEC analyses were performed using Agilent 390‐LC multi‐detector suites. The system was equipped with a PL‐AS RT/MT autosampler, an aPLgel 5 μm guard column and two PLgel 5 μm Mixed D columns (with an exclusion limit of 4,000,000 g/mol). Agilent GPC software was used to analyze the collected data. The eluent was CHCl_3_ with 2% trimethylamine (TEA). A flow rate of 1 mL/min and an injection volume of 100 μL were applied. The column sets were maintained at ambient temperature.

For calibration, refractive index detector 4 capillary viscometers and a dual angle (15° and 90°) light scattering detector were used. The inter‐detector delay was calibrated using a single PMMA narrow standard (Mp 90,250 g/mol) of known concentration. PS EasiVial standards (162–508,000 g/mol, Aglient) analyzed at known concentrations were used for column calibrations. A minimum of 9 points were fit to a third‐order calibration curve.

#### Nuclear Magnetic Resonance (NMR)

All ^1^H NMR (400 MHz), ^13^C NMR (100 MHz), and 2D ^1^H‐^13^C HSQC NMR spectra were recorded using a Bruker Avance 400 spectrometer at 298 K. Approximately 10‐mg samples for ^1^H NMR and 60‐mg samples for ^13^C NMR of the copolymers were dissolved in 0.8 mL CDCl_3_ (with silver foil, 99.8%, Cambridge Isotope Laboratories) and then transferred to a 5 mm‐diameter sample tube. The spectra were calibrated using the residual solvent (CHCl_3_) signals, 7.26 ppm for ^1^H‐NMR and 77.0 ppm for ^13^C‐NMR. The conversion was determined based on the resonance signal intensities comparison of the raw product of unconverted monomers to that of the converted monomers. The amount of incorporated inimer was obtained from the purified copolymer samples. (δ_ɛCL_ = 1.84 ppm, δ_PCL_ = 1.36 ppm, δ_αOHγBL_ = 4.48 ppm, δ_pαOHγBL_ = 5.44 ppm, δ_αOHβMe2γBL_ = 1.07 ppm, δ_αOHβMe2γBL_ = 1.11 ppm).

#### Differential Scanning Calorimetry (DSC)

The thermal properties of the copolymers and homopolymers were measured using DSC equipment (Mettler Toledo DSC 820 Module). Copolymer samples (∼4 mg) were weighted and sealed into 40 µL alumina crucibles. The temperature program was set as (I) heat from −70 to 100 °C, (II) cool down to −70 °C, then (III) heat for a second time to 100 °C. Both heating and cooling rates were set to 10 K/min under a nitrogen atmosphere using a flow rate of 50 mL/min. The melting temperature (*T*_m_) was considered as the maximum value from the melting peaks and was collected from the second heating scan.

## RESULTS AND DISCUSSION

A one‐pot, inimer promoted ROCP‐strategy was developed for the synthesis of branched aliphatic polyesters with pendant hydroxyl groups. The commercially available and biobased 5‐membered lactone, αOHγBL, was chosen to introduce the concept of using 5‐membered lactones in branched polymers and hence to facilitate statistical branching and also to avoid the multistep synthesis generally associated with the preparations of inimers used. The multibranching ROCP system kinetics were studied in detail to explore whether good control over the Sn(Oct)_2_‐catalyzed ROCP for the inimer‐monomer system could be achieved and to determine if the chemical and physical properties of the resulting materials are tunable.

### Initiation Efficiency of the Secondary Alcohol of αOHγBL

αOHγBL is a 5‐membered lactone with a secondary alcohol group on the α‐position and should therefore most likely function as an inimer. To elucidate the efficiency of the secondary alcohol and how the addition of a primary alcohol co‐initiator influences the inimer (αOHγBL)‐ɛCL comonomer system, a number of reactions using BnOH as a co‐initiator was performed. Sn(Oct)_2_ catalyzed ROP is considered a controlled process.^35^, ^36^ The reactions are performed using an initiator, which is typically either a primary alcohol or an amine.^37^ The five‐membered inimer αOHγBL was used here has a secondary alcohol. Like secondary alcohols in many other reactions, it may not be an effective initiator because the propagating chain end is a primary alcohol, which is comparably more active. However, recent publications have shown good efficiency and control over ROP for cyclic esters initiated by secondary alcohols even possessing high steric hindrance.^38^, ^39^

The non‐existing homopolymerization of αOHγBL (entry **1**) verified that γBLs do not undergo homopolymerization under the applied conditions (Table 1). In these systems, αOHγBL may have dual functions. The hydroxyl group could act as an initiating site for the polymerization of lactones, and the γBL part could open and incorporate into a copolymer under the same conditions. To evaluate these two possibilities, equal amounts of inimer and ɛCL were charged in reaction **2** and reaction **3,** but with the addition of BnOH as a co‐initiator in reaction **3**. A drop in *M*_n_ was observed with the addition of a co‐initiator. As the amount of the co‐initiator was increased (**4**), *M*_n_ continued to drop. Similar results were found for reactions performed in solution (**5**, **6**), for which the molecular weight was halved when the co‐initiator was added. A BnOH‐initiated homopolymer (**7**) was conducted as a reference, and a *M*_n_ close to the theoretical value (11,400) of a linear polymer was observed. Therefore, the *M*_n_ and the Ð of the αOHγBL‐ɛCL system are highly influenced by the addition of a co‐initiator. The difference in polydispersity between **3** and **7** provides a hint of the branch formation. The two samples shows small differences in molecular weight but the change in Ð is more dramatic. The inimer initiated chains have the ability to undergo ROP again which can be regarded as a condensing reaction and contributes directly to the increase of Ð. For the pure αOHγBL‐ɛCL system (**2)**, no unconverted monomer signals were distinguishable in the raw ^1^H NMR. If all the αOHγBL acted purely as an initiator, a M_n_ of ∼3,500 is expected. However, the M_n_ of the final product was much higher than the theoretical value. We therefore assume that the high molecular weight is due to the ring‐opening of γBL, which acted as a branch unit and hence that the secondary alcohol in the inimer initiates polymerization.

**Table 1:** Copolymerizations of αOHγBL‐ɛCL With and Without the Co‐Initiator BnOH with Sn(Oct)_2_ ([Sn(Oct)_2_]:[CL + inimer] = 1:400) as a Catalyst in Bulk or Toluene

No.	ɛCL/eq	αOHγBL/eq	BnOH/eq	[ɛCL+αOHγBL]	F (αOHγBL)	M_n_/g·mol^−1^	Đ
1	/	1	/	Bulk	/	/	/
2	1	0.033	/	Bulk	0.032	27,500	3.1
3	1	0.033	0.01	Bulk	0.032	15,700	2.6
4	1	0.033	0.033	Bulk	0.028	10,800	1.6
5	1	0.1	0.01	1 M	0.074	3,200	1.6
6	1	0.1	/	1 M	0.077	7,000	1.6
7	1	/	0.01	Bulk	/	12,800	1.3

F was calculated as the inimer content in the unpurified samples determined by crude reaction mixture (see Supporting Information Figure S2 and S3 for detail calculation). *M*_n_ was obtained from SEC with RI detector using CHCl_3_ as eluent

### ROCP Of inimer‐ɛCL Under Controlled Reaction Conditions

**Scheme 1:**
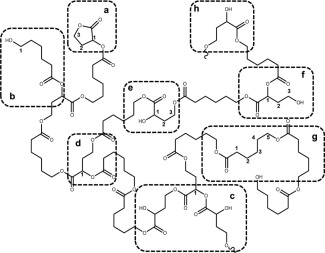
Proposed reaction mechanism and branched polymer structures for a system of αOHγBL and ɛCL. Reactions were performed in bulk or in 1 M toluene solution using Sn(Oct)_2_ as catalyst at 110 °C.

The low ring‐strained γBL requires the addition of a higher ring‐strained lactone to incorporate itself into a polymer chain because γBL cannot homopolymerize to form high molecular polymers. The coordination‐insertion copolymerization of γBL‐ɛCL catalyzed by Sn(Oct)_2_ can be described as a “locking‐in” reaction.^26^ When the incorporation of ring‐opened γBL appears at the propagating chain end, due to its inherent higher tendency of ring‐close again, a more reactive monomer sequence needs to be covalently bonded to incorporate the γBL in the chain to prevent subsequent ring‐closure. In this case, ɛCL comonomer has a higher activity and functions as a lock. After the ɛCL is fully consumed, no additional γBL is available to further increase the γBL content in the polymer, and the chain‐end ring‐opening γBL closes to resume the cyclic form.^26^ The chemical structure of αOHγBL (Scheme 1) shows that when the lactone bond is opened, two hydroxyl functionalities are formed and coordinated with the Sn(Oct)_2_. Both should be available for the initiation or propagation site for ROCP, and thus the opened αOHγBL acts as the branch point. A branched structure will form if sufficient amounts of the αOHγBL are incorporated. The incorporation of the inimer was verified by MALDI‐TOF MS of purified samples from entry **17** (see Supporting Information Fig. S1).

The broad Ð for all samples in Table 1 except for the reaction purely initiated with BnOH, indicates that, aside from the single ROP process, simultaneous self‐condensing polymerizations occur. The key factors influencing the *M*_n_ and Ð are the monomer concentrations ([ɛCL+αOHγBL]), the amount of inimer in the feed, and the reaction time.^40^ All these factors were taken into consideration. First, the effect of the amount of αOHγBL in the feed was examined by gradually increasing the ratio of ɛCL to inimer while maintaining all other reaction conditions the same (**8**, **10**, **11**, **13**, **17**). These reactions exhibited a relatively high conversion of αOHγBL with undistinguishable unreacted inimers after 18 h. The *M*_n_ of the samples increased when the amount of αOHγBL in the initial feed was decreased. This was expected from a ROCP, as αOHγBL itself is not only a comonomer but also an initiator. Higher concentrations of αOHγBL in the feed significantly decreased the molecular weight based on the amount of hydroxyl initiating groups introduced into the polymerization. However, the ability of cyclic γBL to undergo ROP and the incorporation of the γBL moiety into the linear main chain compensated for the reduction to a certain extent.

Second, the systems' dependence on the reaction time and catalyst amount was elucidated. For these one‐pot branched polymer synthesis systems, longer duration reactions are expected to yield higher molecular weights and broader Ð (due to the nature of the Sn(Oct)_2_ catalyst), which can intensify the transesterification process over time.^41^, ^42^ Yu et al. investigated the homopolymerization of a cyclic inimer 6‐hydroxymethyl‐1,4‐dioxan‐2‐one (6‐HDON) catalyzed by Sn(Oct)_2_.^40^ In their study, only when the catalyst to monomer ratio was fixed to 1/400 was a high molecular weight polymer formed. When the ratio was increased to 1/100 or decreased to 1/800, neither ratio afforded a high molecular product. However, no similar phenomenon was observed in our system. When the catalyst to monomer ratio was decreased to 1/1000, polymers with similar molecular weights and dispersities were obtained after 18 h compared to systems using a ratio of 1/400. In addition, in the previous study, slightly prolonged reaction times favored higher molecular weights. Thus, the reaction times were increased to explore if the same trend applied to our system. For entry **9** (0.01 eq inimer, 1 h), a high molecular weight polymer with a low Ð was formed after only one hour with a ɛCL conversion of over 78%, revealing that the hydroxyl group in the αOHγBL can act as an efficient initiating site. A clear polymer melt was formed after approximately 40 min, which indicates that the copolymerization was a very fast process. After another 17 h (**8**, 0.01 eq inimer, 18 h), an even higher molecular weight and a significantly broader Ð were achieved. This large increase in Ð again indicates the characteristic self‐condensing process. The same trend applied to all other reaction pairs (**11**‐**12**, **13**‐**14**, **17**‐**18**‐**19**).

To determine whether the αOHγBL motif in similar monomers also acts as an “inimer” and how other substituents on the inimer effect the reaction, αOHβMe_2_γBL was studied (**20**, **21**, **22**, **23**). From the ^1^H NMR analysis of the raw samples, αOHβMe_2_γBL exhibited a much lower conversion than αOHγBL under equivalent reaction conditions, for which the highest conversion of αOHβMe_2_γBL was 53% compared to the quantitative conversion of αOHγBL. As the inimer feed ratio was increased, the inimer conversion significantly decreased. Both inimers have secondary hydroxyl groups at the α‐position, and thus there should not be a large difference in the initiating efficiency of this secondary alcohol. However, the two methyl groups at the β‐position of αOHβMe_2_γBL introduced steric hindrance to the structure, making the hydroxyl‐group less accessible and less reactive. In addition, the two methyl groups weaken the reactivity of the γ‐lactone bond, which could be due to the Thorpe‐Ingold effect.^43^ When the two protons on a methylene group are substituted by two methyl groups, the steric repulsion between the two methyl groups enlarges the bond angle between them, thereby decreasing the bond angle of the other two substitutes groups. As a consequence, the introduction of two methyl groups to the αOHγBL moiety significantly increases the tendency of ring closure, which converts the opened αOHβMe_2_γBL back to the cyclic form, thus contributing to the low conversion of αOHβMe_2_γBL and the formation of a linear structure (see Supporting Information Fig. S4; Table 2).

**Table 2:** Summary of the Inimers‐ɛCL Copolymerization Catalyzed by Sn(Oct)_2_ With Varied Inimer/ɛCL Feed Ratios

No.	ɛCL/eq	Inimer/eq	F (inimer)	[ɛCL+αOHγBL]	Time/h	*M*_n_/g·mol^−1^	Đ
8	1	0.01	0.010	Bulk	18	47,200	3.0
9	1	0.01	0.010	Bulk	1	34,000	1.5
10	1	0.02	0.019	Bulk	18	32,700	2.9
11	1	0.05	0.048	Bulk	18	28,500	2.9
12	1	0.05	0.048	Bulk	1	11,800	2.1
13	1	0.1	0.091	Bulk	18	27,000	2.7
14	1	0.1	0.091	Bulk	1	5,400	1.9
15	1	0.1	0.091	4 M	18	20,400	2.1
16	1	0.1	0.079	1 M	60	7,900	1.7
17	1	0.2	0.140	Bulk	18	23,500	1.6
18	1	0.2	/	Bulk	1	1,300	1.1
19	1	0.2	0.120	Bulk	100	21,600	2.2
20	1	0.02	0.010	Bulk	18	33,400	1.4
21	1	0.05	0.018	Bulk	18	22,600	1.7
22	1	0.1	0.023	Bulk	18	15,100	1.6
23	1	0.2	0.020	Bulk	18	12,900	1.6

The inimer used in entries 8‐19 was αOHγBL. The inimer used in entries 20‐23 was αOHβMe_2_γBL. F was calculated as the inimer conversion determined by ^1^H NMR on the crude reaction mixture (see Supporting Information Figure S2 and S3 for detail calculation). M_n_ was obtained from SEC with RI detector using CHCl_3_ as eluent

The *M*_n_ determinations presented are based on SEC analysis with conventional RI detector which is known to over‐ or underestimate the true values due to the difference in hydrodynamic volume of the measured samples compared to the linear polystyrene standard used for calibration. Because the separation of polymers in SEC is based on hydrodynamic volumes, any change in the chain architecture also affects the molecular weight values.^44^ Therefore a SEC with triple detectors was used to observe the trend in molecular weights and Đ and to provide additional information on the chain structure of the branched polymers. According to the Einstein viscosity law in which K is a constant independent of chain structure, [*η*] = *KV*_h_/*M*, the intrinsic viscosity [*η*] (IV) positively correlates to the hydrodynamic volume, *V*_h_. Thus, a branched polymer has a lower IV than a linear polymer.^45^

**Figure 1:**
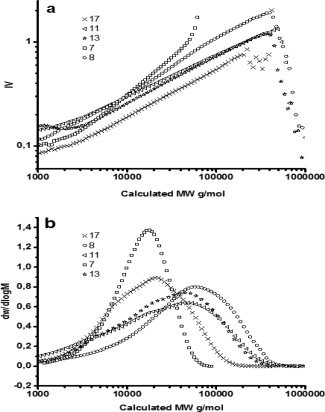
Mark‐Houwink‐Sakurada plots of IV vs the calculated Mw (1a) and the calculated weight distributions vs Mw (1b). The results were obtained from a triple detector SEC using CHCl_3_ with 2% TEA as an eluent.

The results determined from the triple detector SEC are direct evidence of the branched structures of the selected samples, where the degree of branching increases with increasing amount of inimer, and **7** is the linear PCL (Fig. 1). When the calculated M_w_ exceeds 10,000 g/mol, a decrease in intrinsic viscosity is clearly observed, and lower IV values correspond to polymers with higher DB values. Another important parameter used to describe the chain structure is the Mark‐Houwink‐Sakurada exponent *α* which expressed in [*η*]=*KM*_w_^α^. For a linear polymer sample in a good solvent, an *α* value between 0.5 and 0.8 is expected.^46^ For a randomly branched polymer in a good solvent, the value ranges from 0.33 to 0.5. The linear PCL (**7**) has an *α* value of 0.74, whereas branched structures have values between 0.42 and 0.49, Table 3. This result again confirms the randomly or statistically branched structure of the polymers. The differences in molecular structures between the branched and linear polymers can also be described by Zimm branch factor g'.^47^ The intrinsic viscosities of the branched and linear polymers are [*η*]_branched_ and [*η*]_linear_, respectively, and *g*'=[*η*]_branched_/[*η*]_linear_. For linear polymers, *g*' = 1 and *g*' decreases with the increase of branching.^48^

**Table 3:** Summary of the Results Obtained From the Triple Detector SEC Using CHCl_3_ with 2% Trimethylamine (TEA) as Eluent

No.	ɛCL	αOHγBL	*M*_n_/g·mol^−1^	*M*_w_/g·mol^−1^	Đ	α	g'
**7**	1	0	10,100	17,000	1.7	0.74	1.00
**8**	1	0.01	11,600	72,000	6.2	0.50	0.83
**11**	1	0.05	5,400	48,100	8.9	0.42	0.61
**13**	1	0.1	9,700	46,500	4.8	0.44	0.54
**17**	1	0.2	8,400	24,600	2.9	0.46	0.48

All samples with the same numbers as in Table 1 and Table 2 are from the same batches

### Determination of the Degree of Branching

**Figure 2:**
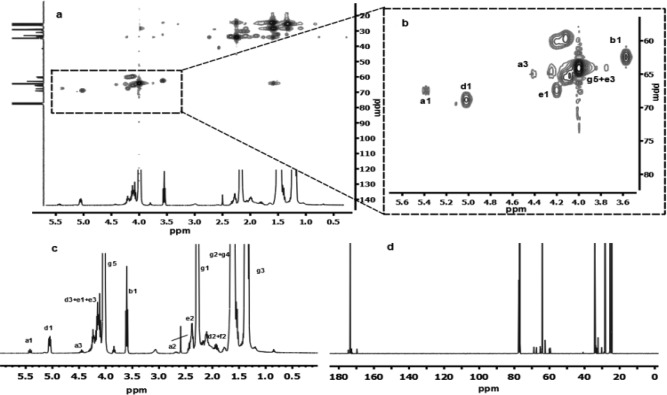
NMR spectra of entry 13 (αOHγBL: ɛCL=0.1:1) catalyzed using Sn(Oct)_2_ in bulk at 110 °C for 18 h. 1a‐ full ^1^H‐^13^C HSQC spectrum; 1b‐ enlarged area for signal assigning, signal labels are correlated to the labels in Scheme 1; 1c‐ ^1^H NMR, signal labels are correlated to the labels in Scheme 1; 1d‐ full ^13^C NMR spectrum.

The degree of branching (DB) is a crucial parameter of branched structures and is used to quantitatively describe how well the branched structure is formed.^49^ One equation to calculate DB in similar inimer promoted systems as well as other branched systems is DB=2D/(2D+L). In this equation D is the number of branch units, and L is the number linear units. In our case, both ^1^H NMR and ^1^H‐^13^C HSQC NMR were utilized first to assign the signals of this novel inimer and subsequently for DB determination (Fig. 2). The possible moieties in the polymer are illustrated in Scheme 1, and the proton signals used to determine DB are marked.

Since D (in DB=2D/(2D+L)) represents the branch site, in which unit **c1** and unit **d1** (corresponding to the marks in Scheme 1) were used, and therefore unit **a**, unit **e**, unit **f** or unit **h** was not considered. For the entry with the lowest amount of inimer, that is, 0.05 eq (**11**), a DB of 0.049 was calculated (additional determination and calculation regarding DB can be found in Supporting Information Figs. S5 and S6). The deviation in the obtained polymers from the ideal structure is primarily due to the existence of unit **a** and unit **e**.

The system shown here is a one‐pot copolymerization with both self‐condensing featured branching and initiation from the secondary alcohols. The DB of the final product is largely dependent on two major factors: the amount of comonomer incorporated and the reactivity of the branching site generated from ROCP. Based on the proposed reaction mechanism, one should expect that more branch centers are formed when more of the αOHγBL inimer is fed into the system. However, because the lactone inimer is a five‐membered ring, its incorporation is limited. As described, ring‐opened γBL must have a ɛCL sequence to “lock” it into the polymer chain. When the feed ratio of the inimer is increased, the relative amount of ɛCL is not sufficient for “locking in”, especially during the later stage of the polymerization when very little of the unconverted ɛCL exists in the system. If a small amount of αOHγBL was initially charged, a higher conversion of γBL to the branch site occurs, but then, due to the small total amount of the branch sites, the branching of the polymer is highly limited. Therefore, 6 polymers with reasonable amounts of αOHγBL and varied reaction conditions (**5**, **6**, **11**, **13**, **17**, **19**) were selected to study the DB features of our system.

The use of a solvent does not favor branching. Samples with different DB values were obtained when the systems were diluted from bulk by toluene to a 1 M monomer concentration in the feed. When 0.1 eq of inimer in 1 M toluene (**6**) was used, a DB of 0.064 was found, whereas when 0.1 eq inimer in bulk (**13**) was used, a DB of 0.084 was determined, which is due to the different polymer aggregation states. In bulk (**13**), the polymer chains are closely packed in the melt after high monomer conversion. This favors the ring‐opening of the unit **a** and transesterification reactions. The pendent primary and secondary hydroxyl groups have a higher chance of forming branch centers via intermolecular transesterification. When toluene was added as solvent, the chains had a higher mobility, and thus the shuffling process was suppressed. The probability of side‐chain active sites undergoing transesterification into another chain decreased, and more unit **a** remained in the cyclical form. The low M_n_ and low Ð are evidence for this. The addition of a co‐initiator further decreased the DB, as evident when 0.1 eq inimer with 0.01 eq BnOH (**5**) were tested (DB=0.044), which is expected because the co‐initiator dilutes the functional chain end.

At prolonged reaction times, a “chain degradation reaction” occurred (**19,** 0.2 eq inimer). Under identical feed ratios, a system that was allowed to proceed for 18 h (**17,** 0.2 eq inimer) (DB=0.124) compared to a system that was reacted for 100 h (**19)** (DB=0.101) had a much lower inimer conversion and a comparatively lower DB. Similar molecular weight drops for one‐pot branched polymer systems after extended reaction time have been previously reported.^40^

### Kinetic Study of the inimer‐ɛCL ROCP

**Figure 3:**
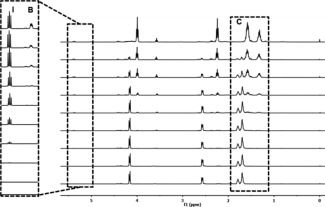
Selected time‐dependent ^1^H‐NMR (CDCl_3_) measurements of ROCP catalyzed using Sn(Oct)_2_ in 1 M toluene solution. Initiation (**I**) correlates to a1 in Scheme **1**, branch formation (B) correlates to unit d1 in Scheme **1**, and ɛCL conversion (C) correlates to unit g2, g3 and g4 in Scheme 1, which are distinguishable by the increasing/decreasing intensity of the signals. The development time (from bottom to top) are 0 min, 5 min, 15 min, 45 min, 90 min, 3 h, 6 h, 10 h, and 24 h.

A kinetic study was performed to elucidate (i) if the inimer‐ɛCL system follows our proposed self‐condensing mechanism, (ii) if the addition of a “dead‐end” co‐initiator can be used to dilute the self‐condensing, (iii) the difference in the reactivity between the two inimers. We investigated three inimer‐ɛCL combinations: (**i**) αOHγBL‐ ɛCL, (**ii**) αOHγBL‐ ɛCL‐ BnOH and (**iii**) αOHβMe_2_γBL‐ ɛCL. From the time of the development of the signals (I) originating from ɛCL initiation, a fast conversion of the αOHγBL appeared upon heating (Fig. 3). During this stage, most of the –OH functional groups on the inimer opened the ɛCL and transformed the secondary –OH group to a primary propagating chain‐end. Branches (B signals) began to emerge after approximately 3 h, and the ratio of B/I gradually decreased as time passed. This ratio exhibited a growing transformation of inimers from cyclic γBL units to dendritic units, hence corroborating the theory of the ROCP self‐condensing mechanism.

**Figure 4:**
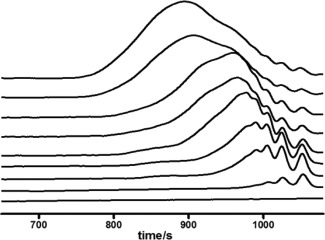
Elugram of the molecular weight and Ð development measured by SEC (RI detection), aliquots collected after 0 h, 1 h, 2 h, 4 h, 6 h, 8 h, 10 h, 24 h and 34 h (from bottom to top).

Furthermore, the elugram collected from SEC verified the possible ROCP self‐condensing mechanism in another way (Fig. 4). During the early stages of the copolymerization (1–4 h), the typical distributions of macromers in higher elution volumes were observed. These distributions corresponded to fast αOHγBL conversion (Fig. 3). As the polymerization time was extended, the elution volumes shifted to lower values, indicating the increasing sizes of the polymer chains. Comparing the elution volumes of 10 and 24 h, not only did the peaks shift, but the size distribution of the polymer chains broadened, indicating that both the *M*_n_ and Ð increased with time. This result is unusual for a controlled ROP process because Ð is independent on the development of *M*_n_ over time.^50^ The only plausible explanation for this occurrence is condensation of the chain end cyclic γBL.

The conversion as a function of time curves exhibited the typical behavior of ROP, including an initial linear stage after which a plateau was reached. For the αOHγBL**‐**ɛCL system, over 50% of the αOHγBL was consumed within the first 90 min of the reaction, and in this stage, the ROCP between αOHγBL and ɛCL was expected to occur. When a αOHγBL is converted, the resulting primary or secondary hydroxyl groups are expected to act as initiating sites due to the high amount of ɛCL monomer in the reaction. This trend is suppressed as more of the ɛCL is consumed. As the reaction time was prolonged, a plateau at ∼85% of inimer conversion was reached. A very small difference was observed between the conversion as a function of time for this system and the system that used 0.01 eq of BnOH as a co‐initiator, indicating that the secondary alcohol on the αOHγBL inimer acts as an efficient initiating site for ROCP. However, the differences between the two systems are clearly illustrated in the conversion‐*M*_n_ curves.

**Figure 5:**
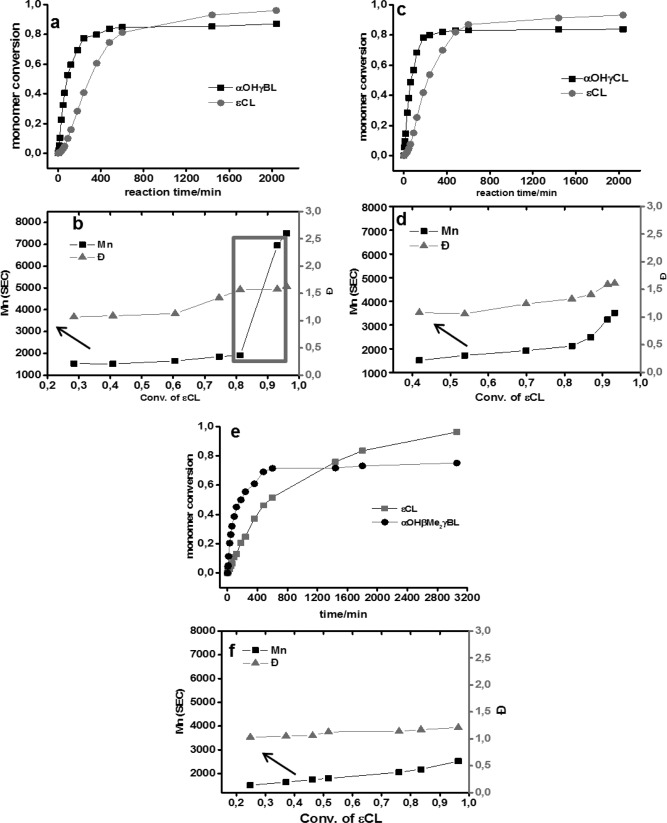
(a, b) Time evolution of the conversion of comonomers 5a αOHγBL:ɛCL = 0.1eq:1eq. The development of Ð and M_n_ versus using SEC with RI detector and the conversion of ɛCL calculated from ^1^H NMR of crude samples. Reactions performed in 1 M toluene solution catalyzed by Sn(Oct)_2_ at 110 °C. (c, d) Time evolution of the conversion of comonomers 5c αOHγBL:ɛCL:BnOH=0.1eq: 1eq: 0.01eq. (e, f) Time evolution of the conversion of comonomers 5e αOHβMe_2_γBL:ɛCL=0.1eq:1eq.

An increase in *M*_n_ was observed in Figure 5(b) after increasing the reaction time from 10 to 24 h, which was not observed in the controlled ROP and is a characteristic feature of self‐condensing polymerization. The αOHγBL inimers first acted as the initiators, and the comonomer ɛCL underwent ROCP from the initiating site. Clusters of macromers with primary hydroxyl and secondary hydroxyl pendent groups were formed during this time. As the reaction proceeded, most of the ɛCL comonomers were consumed, and the conversion of αOHγBL plateaued. The next step was the condensation of the macromers by the ROP of cyclic chain‐end γBLs. The lack of such a pronounced feature in Figure 5(d) further confirmed our assumption. The “dead‐end” co‐initiating site played an important role in the dilution of self‐condensing reactions, as BnOH cannot be opened. Although only 0.01 eq of BnOH was added, the final M_n_ was almost halved compared to Figure 5(b).

For Figure 5(e) (αOHβMe_2_γBL‐ ɛCL), contrasting kinetic features were obtained. The total conversion of this substituted inimer was approximately 75%, and a much slower conversion rate of ɛCL was observed. Interestingly, the linear ɛCL conversion to M_n_ with almost no change in Ð [Fig. 5(f)] is two features that are typical indications of a living polymerization process. Thus, due to the chemical nature of αOHβMe_2_γBL, it cannot act as an inimer, only as an initiator. Based on these comparisons, we concluded the following. First, the αOHγBL‐ ɛCL system follows the suggested self‐condensing scheme after the inimer has reached its equilibrium. Second, the addition of a non‐inimer co‐initiator has a large impact on the system due to the introduction of “dead‐end” chain‐ends. Third, the methyl groups on the beta proton of αOHγBL alter the behavior of the inimer such that αOHβMe_2_γBL does not function as an inimer.

### Thermal Properties of the Branched Copolymers

**Figure 6:**
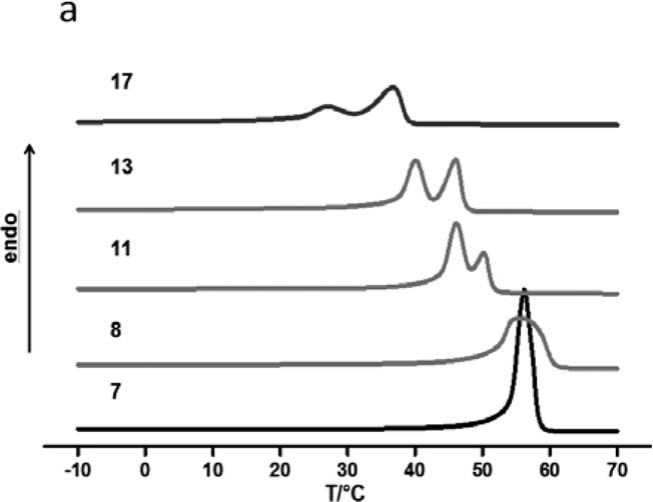
DSC heating traces (**6a**) branched copolymers with different molar fractions of αOHγBL. From bottom to top (αOHγBL:ɛCL, eq:eq): 0:1, 0.01:1, 0.05:1, 0.1:1 and 0.2:1.

The thermal properties of the branched polymers were determined using DSC. All of the samples were obtained from the copolymerization of αOHγBL and ɛCL with varying inimer amounts in the melt at 18 h, and a homopolymer of PCL initiated using BnOH was used as a reference. As shown in Figure 6, a very sharp melting peak with a narrow melting range was obtained for the homopolymer (**7**), which presents the general melting features of a semi‐crystalline material. When BnOH was substituted with the same amount of αOHγBL (**8**), the peak broadened, and the same melting temperature was observed. As a larger amount of αOHγBL was used in the feed and incorporated into the branched polymer, double melting peaks appeared with shifting the *T*_m_s to lower values (Table 4).

**Table 4:** Summary of the Melting Temperatures and Crystallization Temperatures Obtained From the DSC Measurement

No.	*T*_m1_ (°C)	*T*_m2_ (°C)	Δ*T*_m_ (°C)	*T*_c_ (°C)	Δ*H*_c_ (J/g)
**7**	–	55.8 ± 0.2	–	34.0 ± 0.1	92.8 ± 0.8
**8**	–	55.6 ± 0.7	–	28.9 ± 0.2	83,4 ± 0.4
**11**	45.9 ± 0.2	50.3 ± 0.1	4.4	21.8 ± 0.1	74.3 ± 0.4
**13**	39.9 ± 0.1	46.0 ± 0.1	6.1	14.5 ± 0.1	69.6 ± 0.8
**17**	12.3 ± 0.3	26.1 ± 0.1	13.8	−18.5 ± 0.6	46.4 ± 0.3

Results are collected from the second heating scan.

This double melting temperature behavior of the polymers was observed for all the copolymers. Typically, random copolymers display a larger melting temperature range than the corresponding homopolymer of the dominant comonomer. This broadened temperature range is attributed to the varied crystal lamellar thicknesses. A thicker lamellar has a higher T_m_ compared to thinner lamellar. Thus, *T*_m_ differences are due to imperfections or crystalline areas.^51^, ^52^ The double or multiple endothermic peaks may be due to recrystallization during the measurement. At the same cooling rate, copolymers with longer ɛCL blocks form more perfect crystals. The incorporation of γBL and dendritic centers divided the long blocks into shorter sequences and interrupted the crystallization. During heating, some of the imperfect blocks melted first. The chains were then rearranged under elevating temperatures, and an improved crystal structure reformed during the scan. As the temperature increased, the improved crystals melt again, and a second melting peak forms.^53^, ^54^ For low γBL contents, the structure of the polymers can be considered rather as long‐chain branching. The amount of comonomer sequences was not enough to significantly change the crystal reforming, as the dominate ɛCL structure constituted over 99% of the polymer. Therefore, the peak broadened rather than split. When the amount of αOHγBL increased, the γBL units gradually shorten the ɛCL blocks. Thereby, thinner crystals lamellar form, causing a shift in the *T*_m_ toward lowers values. As more dendritic centers form, the difficulty of crystallization during the heating scan increases, which also contributes to a lower *T*_m_.^55^

## CONCLUSIONS

A polymerization pathway utilizing the renewable and commercially available 5‐membered hydroxyl‐functional γBL (αOHγBL) for ɛCL‐based copolyester was successfully designed, yielding statistical branching sites. This work thus provides a straight‐forward one‐pot route to create branched copolyesters while simultaneously extending the current ongoing studies of the 5‐membered γBLs to branched structures. A detailed kinetic study of branching was performed, and it was determined that the 5‐membered lactone was ring‐opened and that a combination of ring‐opening from the secondary alcohol of the γBL and self‐condensation reactions occurred, which both contributed to the DB. For low loading amounts of αOHγBL, complete conversion of the monomer was achieved, whereas lower conversions were obtained at higher loading values. The DB could be varied between 0.049 and 0.124 when 0.05 and 0.2 equivalents of αOHγBL were used, as determined using a combination of ^1^H and ^13^C NMR methods. SEC using a triple detector confirmed the DB trend, both from the decrease in the solution viscosities and the change in α value from 0.74 (linear) to 0.44 (0.1 eq of inimer). The resulting polymers exhibited similar DB values compared to existing branched PCLs. The thermal properties of the copolyesters confirmed the branched structures and followed the expected trend, where a higher amount of branching led to a lower T_m_ due to smaller and more imperfect crystal formation. A second commercially available hydroxyl‐functional γBL αOHβMe_2_γBL was also evaluated, but due to the steric hindrance and deactivation caused by the methyl substitution, it could be determined whether αOHβMe_2_γBL predominately functioned as an initiator.

Unlike many existing inimer‐promoted polymerization methods, which require numerous steps for inimer preparation, αOHγBL greatly simplifies the process. Not only does the method reported here make the production of uniformly branched polyester readily available, but it promotes “green” and sustainable chemistry.

## Supporting information

Supporting Information
