# Supplementary material for: One‐pot inimer promoted ROCP synthesis of branched copolyesters using α‐hydroxy‐γ‐butyrolactone as the branching reagent
Source: J Polym Sci A Polym Chem. 2016 Feb 11;54(13):1908–18. doi: 10.1002/pola.28048 (PMC5067599; doi:10.1002/pola.28048)
Supplement: Supplementary file 1 — Supporting Information [file POLA-54-1908-s001.docx]

Supporting Information

**One-pot inimer promoted ROCP synthesis of branched copolyesters using α-hydroxy-γ-butyrolactone as the branching reagent**

**Geng Hua^1^, Johan Franzén^2^, Karin Odelius^1^**

^1^Department of Fibre and Polymer Technology, KTH Royal Institute of Technology, SE-100 44, Stockholm, Sweden

^2^Department of Chemistry, KTH Royal Institute of Technology, SE-100 44, Stockholm, Sweden

Correspondence to: Karin Odelius (E-mail: *hoem@kth.se*)

Figure S1. MALDI-TOF MS of the purified branched copolymer (αOHγBL: ƐCL =0.2:1)

a. full spectrum and b. zoomed in spectrum

**a**


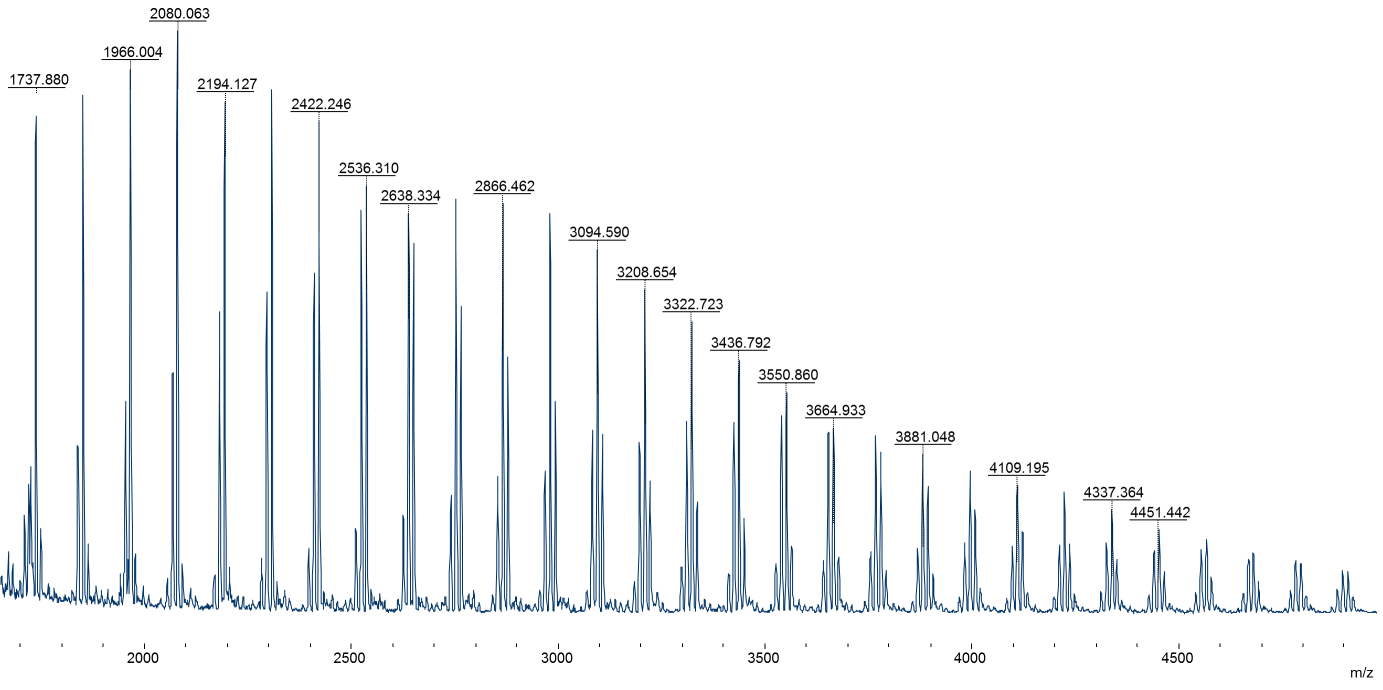

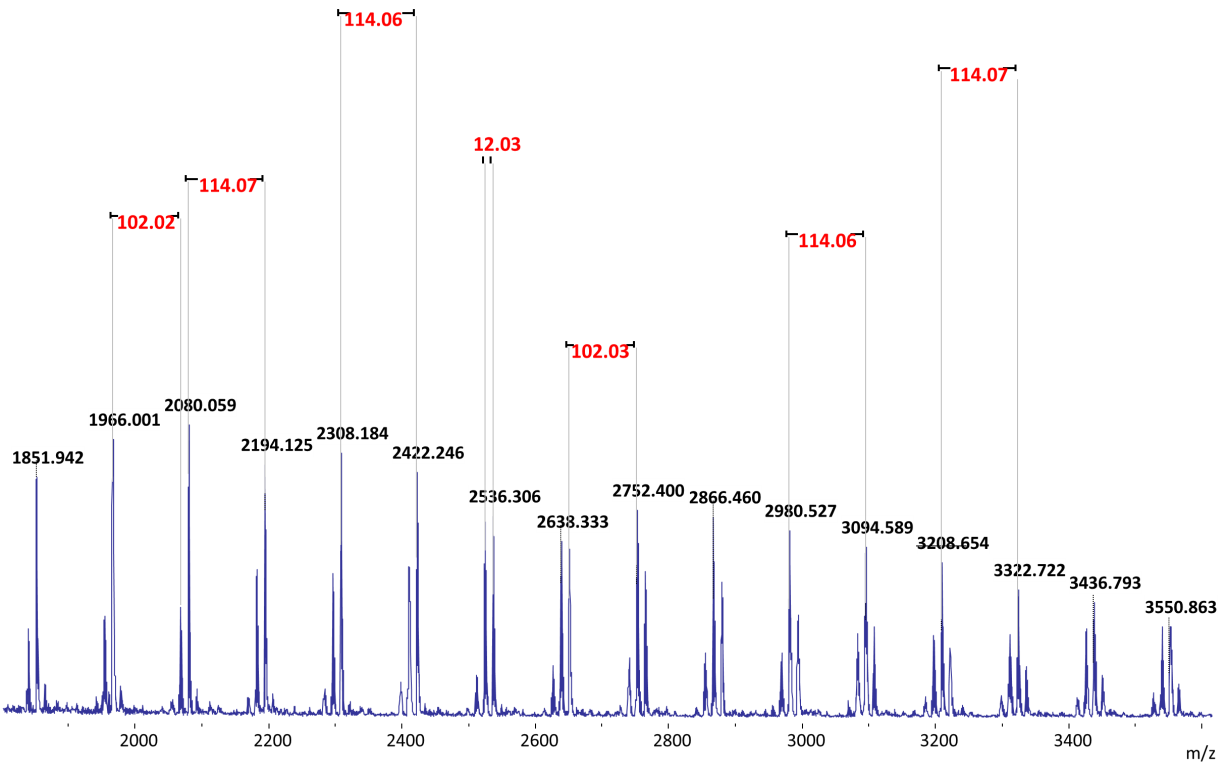


**b**

The sample preparation and experimental procedures for the MALDI-TOF MS are described in a previous article.^1^ The polymer samples are dissolved in THF and mixed together with the matrix (trans-2-[3-(4-tert-butylphenyl)-2-methyl-2-propenylidene] malononitrile) and potassium trifluoroacetate as the ion source.

Take the peak where the mass is 1851.942 for instance:

Mass=a*M_caprolactone_ + b*M_α-hydroxy-γ-butyrolactone_ + 38.96(K^+^ from the ion source)

Mass=15*114.06+1*102.03+38.96=1851.89, found 1851.94.

Figure S2. Stacked ^1^H NMR (αOHγBL: ƐCL =0.1:1) starting material (lower) and raw product (upper) with signals corresponding to the chemical structure. The samples are taken from an entry with αOHγBL: ƐCL=0.1:1 during the middle stage of a polymerization.


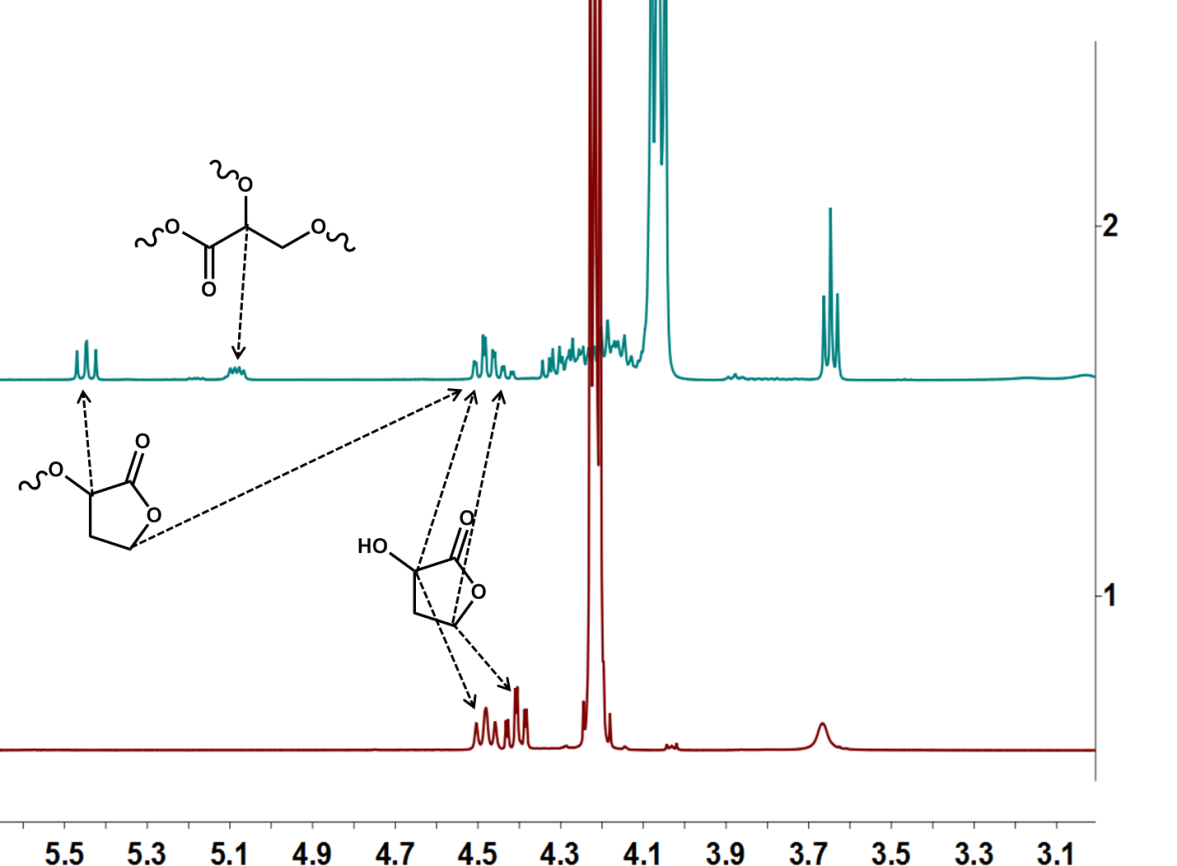


Figure S3. ^1^H NMR of crude product (αOHγBL: ƐCL=0.1:1) for conversion calculation


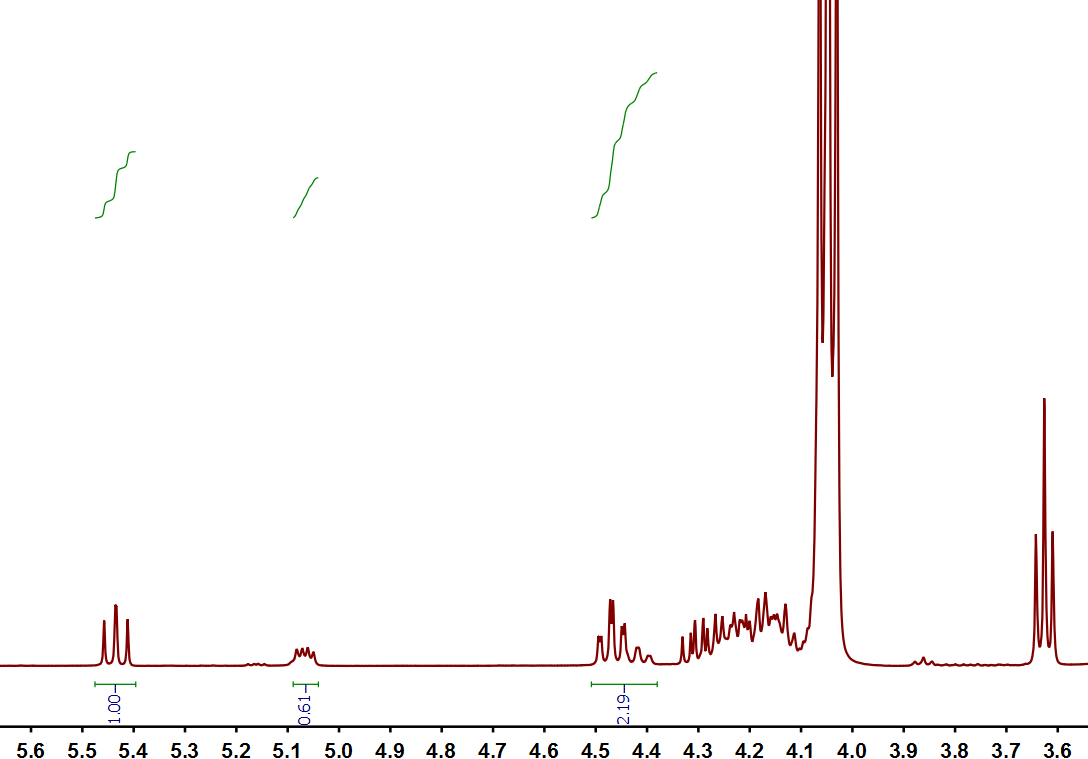


The calculation for the conversion is as following

αOHγBL_converted_%=$\left( 1-\frac{\left[ \alpha OH\gamma BL \mathrm{unreacted} \right]}{\left[ \alpha OH\gamma BL\mathrm{total} \right]} \right)*100\%$=$\left( 1-\frac{\frac{2.19-1}{2}}{\frac{2.19-1}{2}+1+0.61} \right)*100\%$= 73.0%

The sample is taken from the middle stage of an entry with αOHγBL: ƐCL=0.1:1, the content of the converted αOHγBL in the raw sample mixture is

αOHγBL%=$\frac{1}{1+10}$*73%=6.63%

Figure S4. ^1^H NMR of dimethyl substituted αOHγBL initiated linear PCL determined by end group analysis.


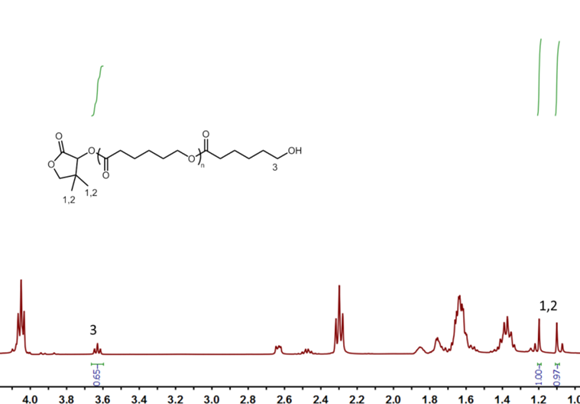


Peaks determination:-C**H**2-OH (3.64, 2H, t) and 1,2 : -C-(C**H**3)2 (1.20, 1.10, 6H, s)

Figure S5. ^1^H NMR of purified product (αOHγBL: ƐCL=0.2:1) before (lower) and after (upper) reacting with trichloroacetyl isocyanate.


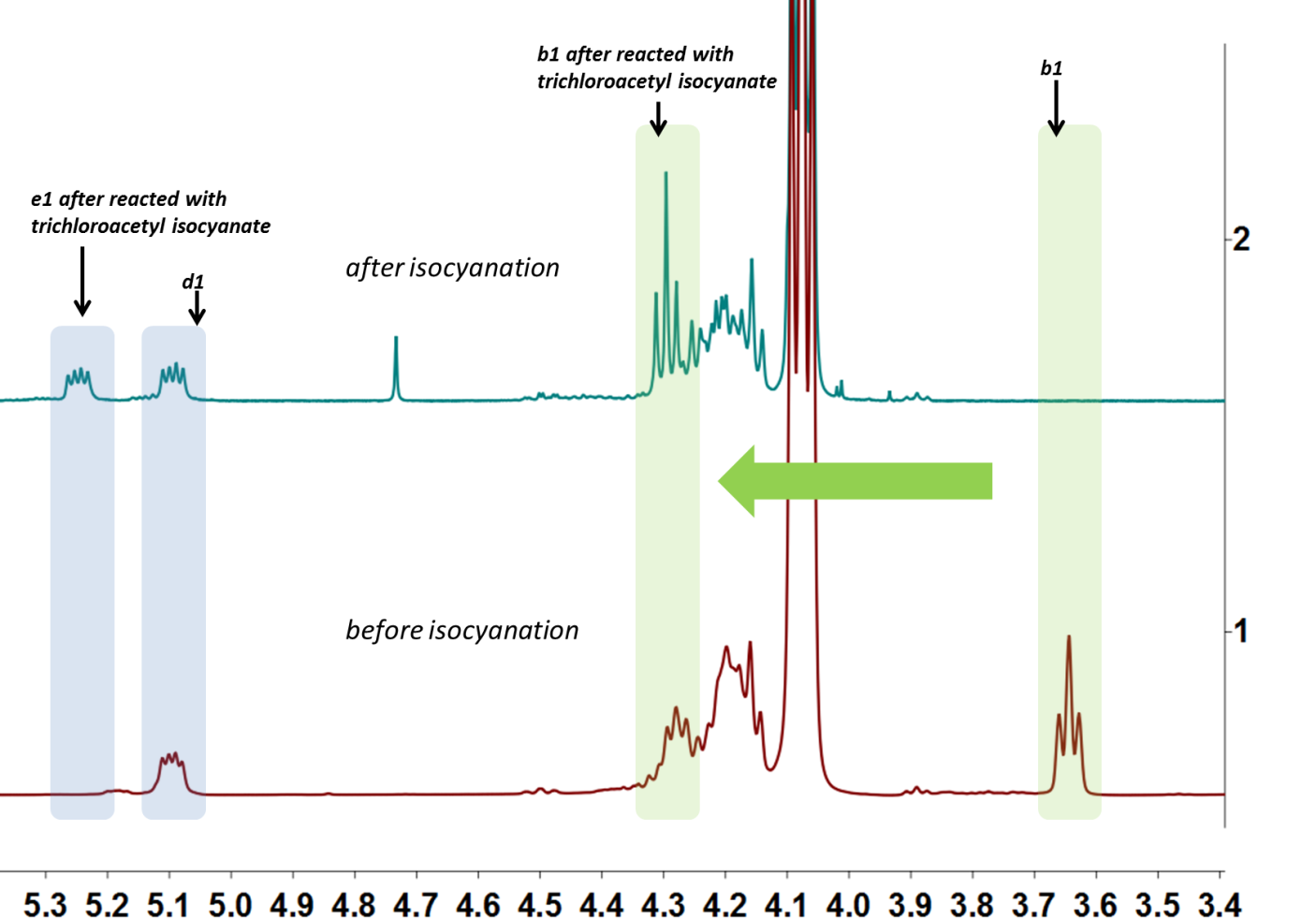


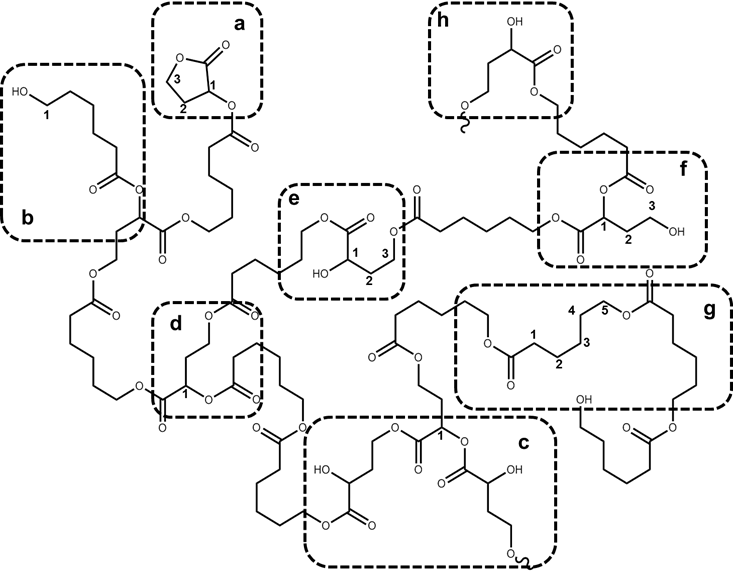


Figure S6. ^1^H NMR of purified product (αOHγBL: ƐCL=0.2:1) after reacting with trichloroacetyl isocyanate for DB calculation.


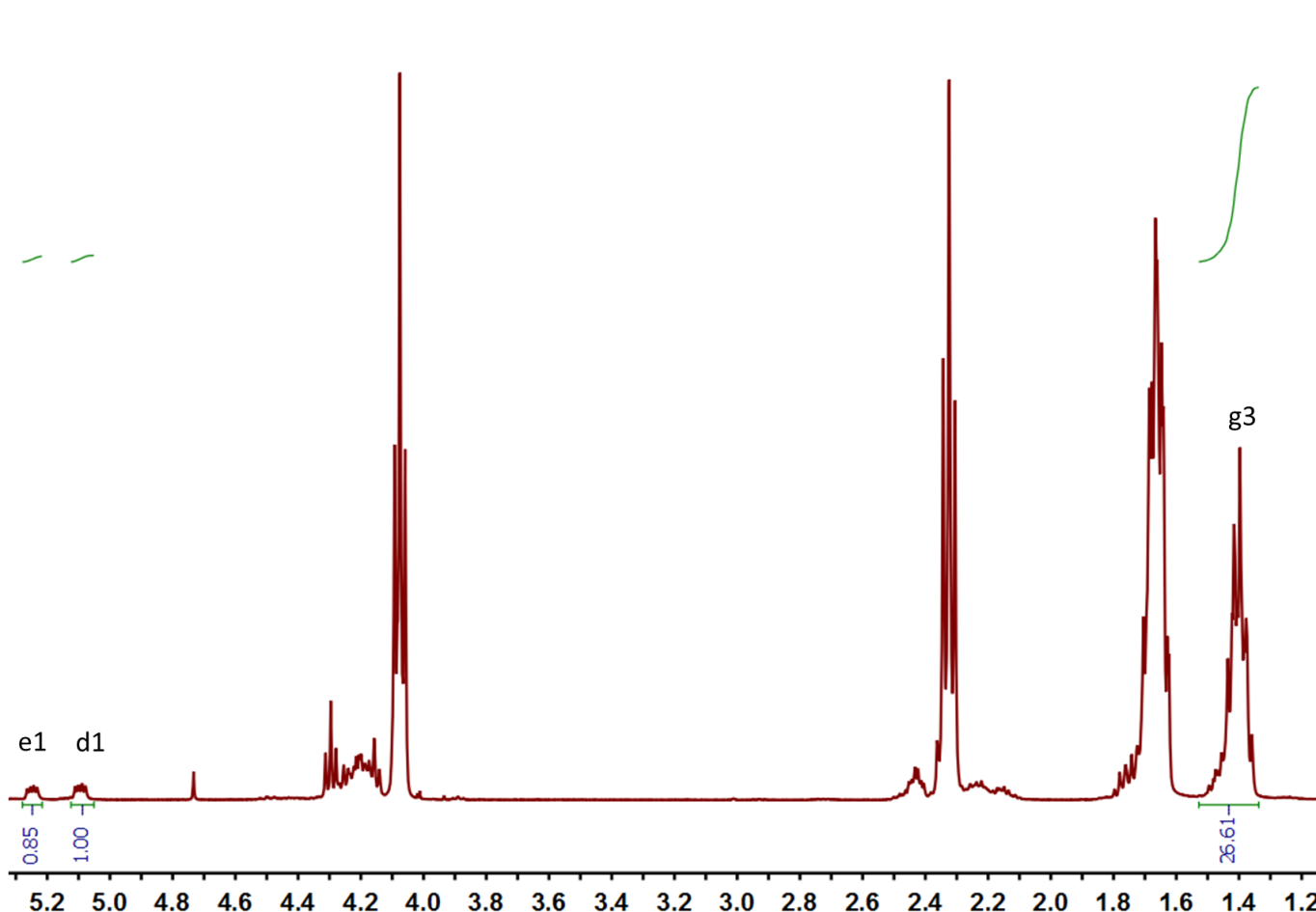


The degree of branching is calculated as following:

$$DB=\frac{2D}{2D+L}$$

where D and L can be obtained from the integral of the corresponding peaks. Take the above sample for instance (sample 17)

$$DB=\frac{2*\boldsymbol{d}\boldsymbol{1}}{2*\boldsymbol{d}\boldsymbol{1}+\boldsymbol{e}\boldsymbol{1}+\frac{\boldsymbol{g}\boldsymbol{3}}{2}}=\frac{2*1}{2*1+0.85+\frac{26.61}{2}}=0.1238$$

The reason that unit g3 (L, 26.61) is divided by 2 is that there are two hydrogens on unit g3.

1. R. Riva, W. Lazzari, L. Billiet, F. Du Prez, C. Jérôme, P. Lecomte. *Journal of Polymer Science Part A: Polymer Chemistry* **2011**, *49*, 1552-1563.
